# Alterations of INPP4B, PIK3CA and pAkt of the PI3K pathway are associated with squamous cell carcinoma of the lung

**DOI:** 10.1002/cam4.191

**Published:** 2014-02-05

**Authors:** Annika Stjernström, Christina Karlsson, Oswaldo J Fernandez, Peter Söderkvist, Mats G Karlsson, Lena K Thunell

**Affiliations:** 1 Division of Cell Biology, Department of Clinical and Experimental Medicine, Faculty of Health Sciences, Linköping UniversitySE-581 85, Linköping, Sweden; 2 School of Health and Medical Sciences, Örebro UniversitySE-701 82, Örebro, Sweden; 3 Department of Vascular and Thoracic Surgery, Örebro University HospitalSE-701 85, Örebro, Sweden; 4 Department of Laboratory Medicine, Örebro University HospitalSE-701 85, Örebro, Sweden

**Keywords:** INPP4B, MAPK pathway, non-small cell lung cancer, PI3K, squamous cell carcinoma

## Abstract

The aim of the study was to investigate how alterations in the PI3K pathway correlate with non-small cell lung cancer subtypes squamous cell carcinoma (SSC) and adenocarcinoma (ADCA). We analyzed copy number variation and protein expression of INPP4B, protein expression of pAkt, PDPK1, and PTEN and mutational status of *PIK3CA* and *PTEN* in 180 cases. Nineteen% displayed loss of *INPP4B* copy, whereas 47% lacked expression, both showing correlation with SCC. Elevated pAkt expression was seen in 63% of all cases, also correlating to SCC. PDPK1 was expressed in 70%, more in male than female patients. Regarding PTEN, 50% displayed loss of expression, of which seven were identified with mutations in the phosphatase domain. We detected nine cases (5%) of *PIK3CA* mutations, all identified as the E545K hot spot mutation in the helical domain, all except one in SCC. When analyzing all PI3K pathway components together, we show that patients with at least one alteration in the PI3K pathway are twice as likely to have SCC, than ADCA. Interestingly, we also found a strong correlation between high pAkt expression and PTEN expression. As comparison, we also analyzed mitogen-activated protein kinase (MAPK) pathway genes, where we identified fifteen *KRAS* mutations (8%) and one *BRAF* mutation (1%), significantly associated to ADCA. No association was found to the Gly972Arg polymorphism of *IRS-1*, involved in activation of both PI3K and MAPK pathways. In conclusion, we show here that several components of the PI3K pathway, alone and in combination, are correlated to development of SCC of the lung.

## Introduction

Lung cancer accounts for 1.2 million new cases annually and is thus the most common cancer worldwide 1. It also represents the most common cause of cancer death with 1.18 million deaths each year. The two important signaling pathways, phosphatidylinositol 3-kinase (PI3K) 2 and mitogen activated protein kinase (MAPK) 3, are both downstream of receptor tyrosine kinases (RTK). They have been studied during several years, and in a wide array of human cancers several components of these pathways are dysregulated. Upon activation of the RTK, PI3K is activated by phosphorylation of either the receptor itself, the intermediate insulin receptor substrate 1 (IRS-1) or by Ras 2. Following activation, PI3K catalyzes the phosphorylation of the inactive phosphatidylinositol-4,5-biphosphate (PI(4,5)P_2_) to phosphatidylinositol-3,4,5-triphosphate (PI(3,4,5)P_3_), both situated in the cell membrane. PI(3,4,5)P_3_ recruits pleckstrin homology domain-containing proteins like Akt and 3-phosphoinositide-dependent protein kinase 1 (PDPK1) to the inner surface of the cell membrane. Thereafter, Akt can be activated through phosphorylation by PDPK1 and the kinase PDPK2. Phosphorylated Akt (pAkt) exerts an immense variety of functions by targeting downstream mediators that regulate processes such as survival and proliferation. The tumor suppressor and phosphatase PTEN is an important regulator and inhibits further signaling by dephosphorylating PI(3,4,5)P_3_ back to PI(4,5)P_2_. In addition, recent evidence indicates that the inositol polyphosphate 4-phosphatase type II (INPP4B) is an important tumor suppressor of the PI3K pathway 4–6. Following the conversion of PI(3,4,5)P_3_ to PI(3,4)P_2_ by SHIP (SH2-containing inositol phosphatase) family members, INPP4B dephosphorylates PI(3,4)P_2_ to PI(3)P, and thus inhibits further signaling. The INPP4B substrate PI(3,4)P_2_ is otherwise also capable of binding pleckstrin homology domains of Akt and PDK1, thereby activating Akt through phosphorylation on Thr308 and Ser473. However, several of the downstream RTK mediators have been found to be altered resulting in a constitutively active signaling pathway. As mentioned, the MAPK pathway is also activated upon RTK activation 2. When the receptor is activated, adaptor proteins and signal-relay proteins will bind to intracellular docking sites and further recruit and activate guanine nucleotide-exchange factors, for example SOS 7. IRS-1 is also involved in activation of the MAPK pathway by interacting with growth factor receptor-bound protein 2 which in turn binds to and translocates SOS 8. Ras is bound to GDP (guanosine diphosphate) in its basal state but will now be able to bind to GTP (guanosine triphosphate), rendering it active 7. Active Ras is able to activate an array of mediators, including Raf and PI3K. Upon stimulation, Raf in turn activates the MEK-ERK (mitogen-activated protein kinase / extracellular regulated kinase) kinase cascade. Like Akt, the ERK family exerts a variety of functions by regulating downstream transcription factors that control processes such as cell survival, growth, and differentiation. GTPase activating proteins (GAPs) are important negative regulators of Ras by increasing the hydrolysis of GTP to GDP rendering Ras back to its basal state. Squamous cell carcinoma (SCC) and adenocarcinoma (ADCA) of the lung respond differently to therapy and there is a growing body of evidence suggesting a separate biology behind the development of the two subtypes 9. In this study, we aimed to investigate how combinations of alterations in important genes of the PI3K and MAPK pathways correlate with different non-small cell lung cancer (NSCLC) tumor subtypes.

## Materials and Methods

### Tissue samples and DNA extraction

This study is based on 180 cases of lung cancer from which tumor tissue was collected at Örebro University Hospital during 1986–1993. The cases consisted of 131 (73%) men and 49 (27%) women with a mean age of 63 (range 36–82). The tissue samples originate from NSCLC constituting of 46% ADCA (among which 57% where men and 43% women) and 51% SCC (among which 89% where men and 11% women). Gender difference with regards to histological subtype was statistically significant; *P *=* *0.000005 (RR = 3.57, 95% CI 1.94–6.56) with women associated with ADCA, while men confer with SCC. Tobacco exposure data were available from 150 (83%) individuals and divided into two groups; former/current smokers and nonsmokers. The study was approved by the regional ethical committee in Linköping. DNA was isolated from frozen sections using Wizard® Genomic DNA Purification Kit (Promega, Madison, WI) according to the manufacturer's protocols. Samples of limited amount were the subject of whole genome amplification using the illustra GenomiPhi V2 DNA amplification kit (GE Healthcare Bio-Sciences AB, Uppsala, Sweden). During 2000–2001, blood samples were collected from healthy participants from the South-East region of Sweden. For *IRS-1*, 488 controls were used, with equal gender distribution and a mean age of 42 years (range 23–78). The participants completed a questionnaire regarding their life style factors including, for example, age, gender, smoking history, and medical history. The study was approved by the regional ethical committee in Linköping. All participants provided informed consent. The DNA isolation of blood samples was performed using QIAamp® DNA Blood Maxi Kit (Qiagen, Solna, Sweden).

### Determination of gene copy number of *INPP4B*

Copy number variation of *INPP4B* was analyzed in a duplex assay together with the reference gene *RNase P*. Real-time quantitative PCR was performed with 25 ng tumor DNA in a 15 *μ*L reaction mixture consisting of 1× of the TaqMan® Genotyping Master Mix, the *INPP4B* assay (assay ID: Hs04806553_cn), and the reference assay (TaqMan® Copy Number Reference Assay *RNase P*), all supplied by Applied Biosystems Inc., Foster City, CA. The *INPP4B* probe was coupled to the reporter FAM (fluorescein amidite) and to a nonfluorescent quencher and the *RNase P* probe was coupled to the reporter VIC and the quencher TAMRA. An absolute quantification assay was performed in the 7900HT Fast Real-Time PCR system (Applied Biosystems Inc.) using the following thermal cycling conditions: 95°C for 10 min followed by 40 cycles of 95°C for 15 sec and 60°C for 60 sec. Samples were run in triplicates in one MicroAmp® Fast Optical 96-well plate along with triplicates of the nontemplate control. On each plate two calibrator samples were included; a two-copy sample from a healthy control and a one-copy sample from a penis cancer case. Both samples had previously been determined in the laboratory by microarray (data not shown). Results were analyzed with a *C*_T_ threshold at 0.2 using the Applied Biosystems CopyCaller™ software (version 1.0), which computed estimated copy numbers based on the comparative threshold cycle method (ΔΔCt).

### Immunohistochemical analysis of INPP4B, pAkt, PDPK1 and PTEN

A tissue microarray (TMA), of the originally formalin-fixed and paraffin-embedded samples, was sectioned at 4 *μ*m onto Dako ChemMate Capillary gap microscope slides (Dako, Glostrup, Denmark), placed at 60°C for 1 h and subjected to immunohistochemistry as follows. Slides were deparaffinized and antigen retrieval was carried out by microwaving the slides intended for analysis of expression of INPP4B, pAkt and PTEN in Tris-EDTA buffer pH 9 at 650 W for 30 min. The slides for PDPK1 analysis did not require treatment for antigen retrieval prior to staining. A ChemMate DAKO EnVision Detection Kit (Dako) was used for immunohistochemistry according to the manufacturer's instructions, with primary antibody incubation for 25 min at room temperature (monoclonal rabbit anti-INPP4B, EPR3108, 1/50; Abcam, Cambridge, UK; monoclonal rabbit anti-PDK1, Y336, 1/50, Abcam; monoclonal rabbit anti-PTEN, #9559, 1/100, Cell Signaling Technology, Danvers, MA) or incubation overnight (∼16°h) at 4°C (monoclonal rabbit anti-Phospho-Akt, #3787, 1/50; Cell Signaling Technology). For negative control slides, the primary antibody was substituted for Dako ChemMate antibody diluent (Dako). Control sections were included and consisted of muscle tissue (skeletal, heart and smooth) for INPP4B, human breast cancer tissue for PDPK1 and pAkt, and for PTEN, human tonsil and prostate cancer tissue was used. For PTEN, a blocking peptide was used according to the manufacturer's procedures (#1250; Cell Signaling Technology). Antibody specificity of anti-Phospho-Akt has previously been established by Andersson et al 10.

### Scoring of immunohistochemistry

Two independent blinded observers (AS and CK) evaluated the expression of INPP4B, pAkt, PDPK1 and PTEN. INPP4B was graded according to immunostaining of the cytoplasm: no expression, −; low expression, +; intermediate to high expression, ++. pAkt was graded according to cytoplasmic and nuclear expression: no expression, −; low expression, +; intermediate expression, ++; and strong expression, +++. PDPK1 was graded as pAkt but according to immunostaining of the cytoplasm and cell membrane. Tumors were graded PTEN^**−**^ when there was no immunostaining of the tumor cells and considered PTEN^**+**^ when the tumor cells were stained in the cell nucleus and cytoplasm.

### PCR amplification of genomic DNA

Primer sequences and annealing temperatures for *PIK3CA* (exons 9 and 20), *PTEN* (exons 5–8), *KRAS* (exons 2 and 3) and *BRAF* (exons 11 and 15) are published by Andersson et al. 10 and the sequences for the single-nucleotide polymorphism (SNP) G972R of the *IRS-1* gene by Almind et al. 11, all custom-made by Invitrogen, Paisley, UK. The PCR reaction was carried out on a Mastercycler® ep (Eppendorf, Horsholm, Denmark) in a total volume of 20 *μ*L containing: 50 ng of genomic DNA; 200 *μ*mol/L of each dNTP; 1.0 *μ*mol/L of each primer for all except *BRAF* and *KRAS* (2 *μ*mol/L); 2 mmol/L MgCl_2_ for all except *KRAS* (3 mmol/L); 1× PCR buffer (containing 200 mmol/L (NH_4_)_2_SO_4_, 750 mmol/L Tris pH 9.0 and 0.1% Tween 20) and finally, 0.5 units Thermo White Taq DNA polymerase (Saveen & Werner AB, Limhamn, Sweden). In order to avoid amplification of a pseudogene on chromosome 22, exon 9 of *PIK3CA* was amplified in a two-step nested PCR using a touch-down program with annealing temperatures from 64 to 61°C and 1.5 mmol/L MgCl_2_ for 30 cycles, followed by a secondary PCR at 60°C for 25 cycles 10. Both *PIK3CA* exon 20 and *PTEN* exon 5 were amplified in two overlapping fragments to produce sizes optimal for single-strand conformation analysis (SSCA).

### Mutation analysis of *PIK3CA*,*PTEN*,*KRAS,* and *BRAF*

In order to screen for potential mutations in *PIK3CA* (exons 9 and 20), *PTEN* (exons 5–8), *KRAS* (exons 2 and 3), and *BRAF* (exons 11 and 15), radiolabelling for SSCA was performed. For the radiolabelling, a second PCR was performed on MJ Research PTC-200 (GMI, Ramsey, MN) in which 1 *μ*L of the primary PCR product was amplified for 12–15 cycles in the presence of 1.0 *μ*Ci of [*α*-^32^P]-dATP (3000 Ci/mmol) (PerkinElmer Sverige AB, Upplands Väsby, Sweden). Samples were thereafter diluted 1:10 with denaturing loading solution (98% formamide, 9.8 mmol/L EDTA (pH 8.0), 0.098% bromphenol blue and xylene cyanol). Following dilution, samples were denatured at 95°C for 3 min and loaded onto a 6% polyacrylamide gel and/or MDE™ gel (Bio-Rad, Hercules, CA and Cambrex Bioscience Rockland Inc., Rockland, ME, respectively). Fragments were separated at 3–6 W constant power at room temperature for 15–17 h. Gels were transferred to 3 mm paper, dried and autoradiographed for 5–48 h. Shifted fragments were excised, reamplified, and purified for sequencing on a MegaBACE™ 500 (GE Healthcare Bio-Sciences AB). In order to label samples, a DYEnamic™ ET Dye Terminator kit (GE Healthcare Bio-Sciences AB) was used according to the manufacturer's protocols.

### Restriction fragment length polymorphism analysis of *IRS-1*

The digestion was carried out by incubating 15 *μ*L of the PCR product for *IRS-1* for 2 h at 60°C in presence of 15 U of *Bst*N I restriction enzyme (New England BioLabs Inc., Ipswich, MA). The samples were analyzed on a 3% NuSieve (Cambrex Bio Science Rockland Inc.): 1% agarose (Invitrogen) gel containing 0.5 *μ*g/mL ethidium bromide and bands were visualized in UV light. Following digestion, three banding patterns depending on genotype were observed: homozygotes for the G allele (GG) displayed three fragments of 158 bp, 81 bp, and 23 bp; homozygotes for the R allele (RR) resulted in four fragments of 107 bp, 81 bp, 51 bp, and 23 bp; heterozygotes (GR) displayed all five fragments; 158 bp, 107 bp, 81 bp, 51 bp, and 23 bp.

### Statistical analysis

Hardy–Weinberg equilibrium was calculated using *χ*^2^-test. Relative risk (RR), odds ratio (OR), 95% confidence intervals (CI), *P*-values, and *χ*^2^-test for trend were calculated using the StatCalc feature in the Epi Info software package, version 3.5.1 (Centers for Disease Control and Prevention, Atlanta, GA). Statistical significance was considered when *P *<* *0.05.

## Results

### Copy number analysis of INPP4B

We successfully determined the copy number of *INPP4B* in 176 tumor samples displaying the following distribution; 15% ≤1 copies, 62% 2 copies, and 23% ≥3 copies. Statistical analysis revealed a borderline significant association between ≤1 copies of *INPP4B* and SCC (RR = 1.44, CI 95% 1.03–2.01, *P *=* *0.06; Table 1).

**Table 1:** Gene copy number of *INPP4B*, expression of INPP4B, pAkt, PDPK1 and PTEN, mutations of *PIK3CA*,*PTEN, KRAS, BRAF* as well as *IRS-1* G972R genotypes in relation to NSCLC subtype (ADCA and SCC) and gender.

	Total, *N* (%)	ADCA, *N* (%)	SCC, *N* (%)	Men, *N* (%)	Women, *N* (%)
*INPP4B*					
≤1 copies	26 (19)	8 (12)	17 (25)	21 (21)	5 (15)
2 copies	109 (21)	57 (88)	51 (75)	81 (79)	28 (85)
			*P *=* *0.06		*P *=* *0.49
INPP4B					
** −**	81 (47)	24 (30)	55 (38)	67 (53)	14 (30)
**+/++**	91 (53)	55 (70)	33 (62)	59 (47)	32 (70)
			***P *****=***** *****0.00003**		***P *****=***** *****0.008**
pAkt					
−/+	63 (37)	40 (51)	23 (26)	48 (38)	15 (33)
**++/+++**	109 (63)	39 (49)	65 (74)	78 (62)	31 (67)
			***P *****=***** *****0.001**		*P *=* *0.51
PDPK1					
**−**	15 (9)	9 (12)	6 (7)	9 (7)	6 (13)
**+**	36 (21)	16 (20)	20 (23)	26 (21)	10 (22)
**++**	89 (52)	40 (51)	45 (52)	63 (50)	26 (58)
**+++**	30 (18)	13 (17)	16 (18)	27 (22)	3 (7)
			*P =* *0.52		*P **=***** *****0.05**
PTEN					
**−**	87 (50)	36 (46)	48 (54)	69 (54)	18 (39)
**+**	86 (50)	43 (54)	41 (46)	58 (46)	28 (61)
			*P *=* *0.28		*P *=* *0.08
*PIK3CA*					
wt	170 (94)	80 (98)	72 (90)	123 (94)	47 (96)
Mutant	10 (6)	2 (2)	8 (10)	8 (6)	2 (4)
			***P *****=***** *****0.05**^1^		*P *=* *0.46^1^
*PTEN*					
wt	175 (97)	80 (98)	89 (97)	129 (98)	46 (94)
Mutant	5 (3)	2 (2)	3 (3)	2 (2)	3 (6)
			*P *=* *0.55^1^		*P *=* *0.13^1^
*KRAS*					
wt	164 (92)	69 (85)	76 (98)	121 (93)	43 (88)
Mutant	15 (8)	12 (15)	2 (2)	9 (7)	6 (12)
			***P *****=***** *****0.006**		*P *=* *0.25
*BRAF*					
wt	177 (99)	80 (99)	91 (100)	129 (100)	48 (98)
Mutant	1 (1)	1 (1)	0 (0)	0 (0)	1 (2)
			*P *=* *0.47^1^		*P *=* *0.28^1^
*KRAS*/*BRAF*					
wt	162 (91)	68 (84)	89 (98)	120 (93)	42 (86)
Mutant	16 (9)	13 (16)	2 (2)	9 (7)	7 (14)
			***P *****=***** *****0.001**		*P *=* *0.13
*IRS-1*					
GG	149 (88)	71 (91)	73 (84)	104 (85)	45 (94)
GR	21 (12)	7 (9)	14 (16)	18 (15)	3 (6)
			*P *=* *0.17		*P *=* *0.13

ADCA, adenocarcinoma; N, number of individuals; NSCLC, non-small cell lung cancer; SCC, squamous cell carcinoma; wt, wildtype. Statistically significant associations in bold.

* *P*-value for trend.

1 Fisher's exact test.

### Immunohistochemical analysis of INPP4B, pAkt, PDPK1 and PTEN

**Figure 1:**
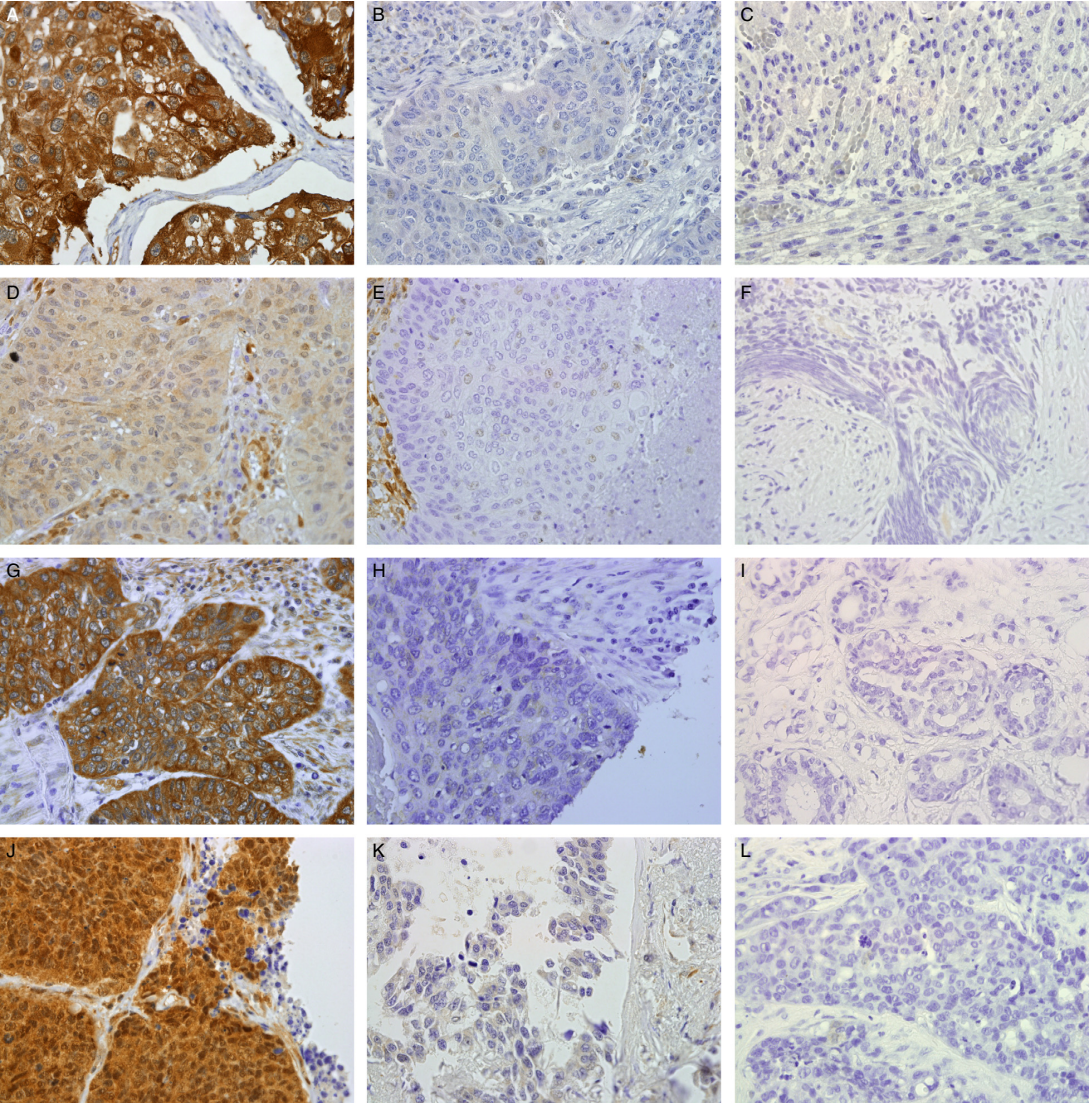
(A–L) INPP4B, PTEN, PDPK1, and pAkt expression and corresponding control tissue. (A) INPP4B positive expression in SCC, graded as ++ showing strong cytoplasmic staining, (B) INPP4B negative expression in SCC, (C) INPP4B negative control in skeletal muscle, (D) PTEN positive expression in SCC, (E) PTEN negative expression in SCC, (F) PTEN negative control with blocking peptide in human prostate, (G) PDPK1 positive expression in SCC, graded as +++ showing strong cytoplasmic and membrane expression, (H) PDPK1 negative expression in SCC, (I) PDPK1 negative control in breast cancer, (J) pAkt positive expression in SCC, graded as +++ showing strong cytoplasmic and nuclear expression, (K) pAkt negative expression in ADCA, and (L) pAkt negative control in breast cancer. Photographs were taken at 40× magnification. ADCA, adenocarcinoma; SCC, squamous cell carcinoma.

Representative photographs of INPP4B, PTEN, PDPK1, and pAkt expression and corresponding control tissue are shown in Figure 1A–L. Expression of INPP4B was established in 172 tumor samples and graded as follows: 47% −, 22% +, and 31% ++. However, for further analysis, the groups with low (+) and intermediate to high (++) expression were merged. Of the 47% of samples displaying loss of INPP4B expression, 19% also show loss of heterozygosity of *INPP4B* (Table 1). Negative expression of INPP4B shows a strong correlation with SCC compared to ADCA (RR = 1.86, 95% CI 1.37–2.52, *P *=* *0.00003 as well as to men compared to women (RR = 1.28, 95% CI 1.06–1.53, *P *=* *0.008). Expression of pAkt was successfully determined in 172 tumor samples graded into the following groups: 0% −, 37% +, 60% ++, and 3% +++. Due to low samples number in the negatively and strongly stained groups, the following expression patterns were formed: −/+ (low) and ++/+++ (high). Sixty-three percent of the NSCLC cases displayed intermediate or strong pAkt expression (Table 1). Relative risk analysis revealed a statistical correlation between SCC and high levels of pAkt expression (RR = 1.71, 95% CI 1.20–2.45, *P *=* *0.001). Status of PDPK1 expression was established in 170 tumor samples with the following distribution: 9% −, 21% +, 52% ++, and 18% +++ (Table 1). Upon analysis of *χ*^2^ for trend for PDPK1 expression between men and women, a significant difference was seen where men confer with higher expression levels of PDPK1 (*χ*^2^ for linear trend 3.87, *P *=* *0.05). No significant observation was observed between SCC and ADCA. PTEN expression was successfully determined in 173 NSCLC tumor samples with 50% showing loss of expression (Table 1).

### Mutational analysis of *PIK3CA* and *PTEN*

Mutational analysis of the hot spots in the helical and kinase domains of *PIK3CA* and the phosphatase domain of *PTEN* was also performed. For *PIK3CA*, of 180 samples analyzed, nine cases (5%) with mutations were found, all displaying the E545K alteration (Table 2). Having a mutation in *PIK3CA* confer with SCC (RR = 1.69, 95% CI 1.19–2.40, *P *=* *0.05; Fisher's exact test; Table 1) compared to ADCA. For *PTEN*, five mutations (3%) were discovered, which coincide with loss of PTEN expression (Table 2).

**Table 2:** Summary of *PIK3CA, PTEN, KRAS,* and *BRAF* mutations.

Gene	Exon	Nucleotide	Codon	Domain	No. of patients
*PIK3CA*	9	GAG > AAG	E545K	Helical domain	9
*PTEN*	5	CGA > TGA	R130X	Phosphatase domain	1
	5	TTA > TAA	L139X	Phosphatase domain	1
	5	CGG > CAG	R142Q	Phosphatase domain	1
	5	Del AAGCT	125-126	Phosphatase domain	1
	6	GGA > GAA	G165E	Phosphatase domain	1
	6	Del AGAA	183-184	Phosphatase domain	1
	8	Del CCCT	319-320	C2 domain	1
*KRAS*	2	GGT → TGT	G12C	GTP binding	7
		GGT → GTT	G12V	GTP binding	3
		GGT → GAT	G12D	GTP binding	1
	Subtotal	GGT → GCT	G12A	GTP binding	1
		GGC → TGC	G13C	GTP binding	3
					15 (8%)
*BRAF*	15	GAT → GCT	D593A	Protein kinase	1
	Subtotal				1 (1%)

### Mutational analysis of *KRAS* and *BRAF*

For *KRAS* exon 2, 180 samples were analyzed and 15 mutations (8%) were found: seven G12C, three G12V, one G12D, one G12A, and three G13C (Table 2). No mutations were found among the 179 samples analyzed for *KRAS* exon 3 or among the 179 samples analyzed for *BRAF* exon 11. One mutation (D593A; 1%) was discovered among the 178 samples analyzed for *BRAF* exon 15 (Table 2). Patients harboring a *KRAS* mutation are significantly associated with the NSCLC subtype ADCA (RR = 1.80, 95% CI 1.37–2.37, *P *=* *0.006; Table 1). For *BRAF* alone, no statistical associations were found (Table 1). However, pooling of *KRAS* and *BRAF* mutations showed that there is a significant association with ADCA compared to SCC when having a mutation in the MAPK pathway genes (RR = 2.00, 95% CI 1.53–2.61, *P *=* *0.001; Table 1).

### Genotyping of *IRS-1 G972R*

One hundred and seventy NSCLC cases and 488 controls were successfully genotyped. The genotype frequencies of the control group were found to be in Hardy–Weinberg equilibrium. Genotype and allele frequencies for *IRS-1* G972R with their OR and corresponding 95% CI are presented in Table 3. There were no significant risk associations observed when comparing the different genotype and allele frequencies, nor when analyzing the association between homozygous wildtypes and the combination of heterozygous and homozygous mutants. Dividing the cases based on NSCLC subtype, gender or according to tobacco exposure did not reveal any statistically significant associations (Table 1; data not shown for tobacco exposure).

**Table 3:** Genotype and allele frequencies of *IRS-1* G972R for cases and controls.

	Cases *N*, (%)	Controls *N*, (%)	OR	95% CI	*P*-value
*IRS-1* G972R					
GG	149 (88)	441 (90)	1	0.77–2.47	0.25
GR	21 (12)	45 (9)	1.38	NA	NA
RR	0 (0)	2 (1)	NA		
GG	149 (88)	441 (90)	1		
GR/RR	21 (12)	47 (10)	1.32	0.74–2.36	0.32
G-allele	319	927	1		
R-allele	21	49	1.25	0.71–2.17	0.41

CI, confidence interval; N, number of individuals; NA, non applicable; OR, odds ratio.

### Correlation of different variables

We were also interested in evaluating possible associations by correlating the expression of INPP4B, pAkt, PDPK1, and PTEN as well as *IRS-1* genotypes and *INPP4B* copy number pairwise. Loss of INPP4B expression correlates with loss of PTEN expression (RR = 1.49, 95% CI 1.10–2.02, *P *=* *0.009; data not shown). Furthermore, PTEN and pAkt expression also displayed a strong association (RR = 0.55, 95% CI 0.41–0.74, *P *=* *0.00008; data not shown) showing that there is a decreased likelihood of being PTEN negative when expressing high levels of pAkt. This is also statistically significant with similar RR values when combining cases being negative for INPP4B and PTEN expression compared to pAkt expression status. However, there is no association between INPP4B expression and pAkt expression (RR = 0.92, 95% CI 0.73–1.15, *P *=* *0.46.

We were also interested in correlating an altered PI3K pathway and a normal PI3K pathway to the NSCLC tumor subtypes ADCA and SCC, gender, smoking status, *IRS-1* G972R genotypes as well as to *KRAS*/*BRAF* status. An altered PI3K pathway was considered when having a mutated *PIK3CA*, mutated *PTEN*, loss of PTEN, loss of heterozygosity of *INPP4B*, and/or increased expression of pAkt (++/+++) and a normal pathway when being wildtype for *PIK3CA*, wildtype for *PTEN*, expressing PTEN, two or more copies of *INPP4B*, and low expression of pAkt (−/+). Patients having at least one alteration in the PI3K pathway show a borderline significant association with SCC (RR = 2.01, 95% CI 0.94–4.30, *P *=* *0.02; Table 4).

**Table 4:** NSCLC subtype (ADCA and SCC), *IRS-1* G972R genotype and *KRAS*/*BRAF* mutations in relation to a normal or altered PI3K pathway.

	Subtype, *N* (%)		*IRS-1* G972R, *N* (%)		*KRAS/BRAF*,*N* (%)	
	ADCA	SCC	wt	Het	wt	Mut
PI3K pathway						
Normal^1^	13 (16)	5 (6)	17 (11)	1 (5)	18 (11)	0 (0)
PI3K pathway						
Altered^2^	67 (84)	85 (94)	134 (89)	20 (95)	140 (89)	15 (100)
		***P *****=***** *****0.02**		*P *=* *0.32*		*P *=* *0.18*

ADCA, adenocarcinoma; het, heterozygous; mut, mutated; N, number of individuals; NA, non applicable; NSCLC, non-small cell lung cancer; SCC, squamous cell carcinoma; wt, wildtype. Statistically significant associations in bold.

* Fisher's exact test.

1 Normal is defined as having following; wildtype *PIK3CA*, wildtype *PTEN*, PTEN, two or more copies of *INPP4B* as well as low expression of pAkt (−/+).

2 Altered is defined as having one of following; mutated *PIK3CA*, mutated *PTEN*, loss of PTEN, loss of heterozygosity of *INPP4B,* and/or increased expression of pAkt (++/+++).

## Discussion

The aim of this study was to further investigate how alterations in the PI3K pathway correlate with NSCLC tumor subtypes. We also stratified for gender and tobacco exposure, as smoking is one of the leading causes of lung cancer 12. We show here that 19% of the cases displayed loss of *INPP4B* copy, whereas 47% lacked expression, both showing correlation with SCC. Elevated pAkt expression was seen in 63% of all cases, also correlating to SCC. PDPK1 was expressed in 70%, more in male than female patients. Regarding PTEN, 50% displayed loss of expression, of which seven were identified with mutations in the phosphatase domain. We detected nine cases (5%) of *PIK3CA* mutations, all identified as the E545K hot spot mutation in the helical domain, all except one in SCC, confirming data from previous studies of similar size 13–15. When analyzing all PI3K pathway components together, we show that patients with at least one alteration in the PI3K pathway are twice as likely to have SCC, than ADCA. This is in line with the emerging evidence of the importance of the PI3K pathway as a possible target for therapy strategies in 20–30% of SCC 9. When taking additional alterations, such as *PIK3CA* amplification and AKT1 mutation, into account this further corroborates the role of PI3K pathway 9,14,16–18. Interestingly, we also found a strong correlation between high pAkt expression and PTEN expression. As comparison, we also analyzed MAPK pathway genes, where we identified fifteen *KRAS* mutations (8%) and one *BRAF* mutation (1%), significantly associated to ADCA. No association was found to the Gly972Arg polymorphism of *IRS-1*, involved in activation of both PI3K and MAPK pathways. In conclusion, we show here that several components of the PI3K pathway, alone and in combination, are correlated to development of SCC of the lung.

As mentioned earlier, recent evidence indicates that INPP4B is an important tumor suppressor of the PI3K pathway 4–6,19. This is based on the fact that loss of heterozygosity at the *INPP4B* locus correlates with ovarian cancer and aggressive hormone receptor-negative basal-like breast cancer as well as poor patient survival. An immunohistochemistry study on prostate cancer has also shown statistically significant downregulation of INPP4B 20 and altered INPP4B has been reported in 8% of primary prostate tumors and 47% of metastases 21. In normal lung tissue, INPP4B is strongly expressed in bronchial respiratory epithelial cells and weakly in pneumocytes 22. Our study is the first to show that loss of heterozygosity at the *INPP4B* locus as well as lack of expression is associated with the NSCLC subtype SCC. Furthermore, *PTEN* has been reported to be mutated in about 5–15% of all lung cancers, for example, rendering the protein truncated or with reduced phosphatase activity 23. In the present study, mutational analysis of *PTEN* showed that five (3%) of the analyzed tumors had a mutation in *PTEN,* equally distributed between SCC and ADCA, all conferring with lack of PTEN expression. On the other hand, a study by Jin et al. 24 showed a higher frequency of *PTEN* mutation in SCC (10.2%) than ADCA (1.7%). Total loss of PTEN expression is more frequent than mutations, ranging between 24% and 44%, and is proposed to be due to not only genetic alterations but also epigenetic alterations such as promoter methylation as well as posttranslational alterations, such as decreased protein synthesis or increased degradation 25,26. Our study could also confirm the high frequency of loss of PTEN expression among NSCLC cases. Although we did not find a difference between SCC and ADCA for PTEN staining, other studies have reported differential PTEN loss in SCC and ADCA; 59% vs 34% and 21% vs 4%, respectively, further strengthening the impact of the PI3K pathway on SCC development in the lung 14,27.

Interestingly, we show that there is a positive correlation between PTEN and pAkt expression; the likelihood of being PTEN negative is decreased when expressing high levels of pAkt (RR = 0.55, 95% CI 0.41–0.74, *P *=* *0.00008). This is on the contrary to what one would expect and to what others have seen. Lim et al. 28 did not find any association between PTEN and pAkt expression and not with age, gender or tobacco exposure either; however, they did show that loss of PTEN or expression of pAkt correlate with more aggressive tumors with poor prognosis. On the other hand, Tang et al. 29 showed that loss of PTEN expression alone confers with a poor prognosis and they could also correlate high levels of pAkt and loss of PTEN to poor differentiation, lymph node involvement, distant metastasis, late stages as well as to poor prognosis which is in accordance with what is expected. As PTEN is an important regulator of the PI3K pathway by converting PIP_3_ back to PIP_2_, loss of PTEN would expectedly result in an increased activity and thus increased expression of pAkt. However, recent research proposes a new pathway for regulation of PTEN; an Akt-EGR1-ARF-PTEN axis where decreased expression of PTEN eventually leads to increased expression of pAkt 30. pAkt in turn phosphorylates the early growth response gene 1 (*EGR1*) transcription factor which after sumoylation by ARF (p14) transactivates the *PTEN* promoter resulting in an increased expression of PTEN. This feedback-loop might explain the relationship we found between PTEN and pAkt. There may of course also be other regulators of pAkt levels in these malignancies and further investigations are necessary. When comparing expression between INPP4B and pAkt, we find no correlation, implying that INPP4B does not regulate phosphorylation of Ser473-Akt in SCC and ADCA.

A limitation of the immunohistochemical analysis is that TMAs consist of small pieces of the tumor core with no or little amount of normal tissue. Therefore, we cannot make a statement of the levels of protein expression in the normal lung tissue. However, with regard to PTEN and pAkt expression, Tang et al. 29 showed that zero out of 20 normal lung specimens expressed pAkt and conversely that 20 out of 20 specimens expressed PTEN, which is in agreement to what is expected to find in normal lung tissue. On the other hand, the expression of PDPK1 has not to our knowledge been studied in lung tissue before, neither normal nor tumor. Therefore, we cannot draw any conclusion of the level of expression or where to make a cutoff for decreased or increased expression. However, PDPK1 expression has been immunohistochemically analyzed in normal ovaries, benign and malignant ovarian tumors 31. The results showed a lack of PDPK1 expression in normal ovaries, weak expression in benign tumors, and elevated expression in ovarian carcinomas which indicate an oncogenic role of PDPK1 in ovarian cancer progression. Another study, focusing on semiquantitative RT-PCR analysis for comparing, among other factors, the expression of PDPK1 between normal and oral SCC revealed an increased expression of PDPK1 in tumors, suggesting an involvement in the pathogenesis of oral SCC 32. Also, *PDPK1* has been shown to be mutated in the kinase domain in colorectal cancer, further suggesting a potential oncogenic role of PDPK1 33. However, Lee et al. 34 were not able to find any mutations in exon 10 of *PDPK1* in a small number of colorectal, gastric, or lung carcinomas. For the future, it would be interesting and relevant to investigate the expression of PDPK1 in normal lung tissue and to further analyse the mutational status of *PDPK1*. However, the findings concerning PDPK1 expression in ovarian and oral tissue, where the tumor tissue has increased expression compared to the normal tissue, is perhaps not surprising since, for example, decreased PTEN expression would lead to an increased expression of PDPK1 and, further on, an increase in pAkt. In our study, if drawing the same cutoff value for PDPK1 as for pAkt, 70% showed overexpression of PDPK1. However, we were not able to find any association connected to the different levels of PDPK1 expression. Perhaps PDPK1 is not an important component of the PI3K pathway in the development of NSCLC.

In lung cancer, activating point mutations of the *KRAS* gene was detected as early as 1987 by Rodenhuis et al. 35. A meta-analysis revealed that ∼20% of all lung cancer cases harbor *KRAS* mutations, and that ADCA more frequently possess *KRAS* mutations compared to SCC, 23% versus 7% 36. The discovery of *KRAS* mutations in 8% of all analyzed cases is less than the average of 20% in lung cancer 36. The distribution of the mutations among the tumor subtypes ADCA and SCC (15% vs. 2%) was also somewhat less than the average distribution (23% vs. 7%) 36. All mutations detected were situated in exon 2, codon 12 and 13, corresponding to the GTP-binding hot spot region. A point mutation, in the hot spot region, leading to an amino acid substitution will decrease the intrinsic GTPase activity of Ras and also confer resistance to GAPs 7. Therefore, GTP-bound Ras will accumulate, rendering the MAPK pathway constitutively activated.

*BRAF* mutations occur primarily in exons 11 and 15 37,38 and the frequency of mutations in human NSCLC is low, 1–3% 37,38, and it is therefore not surprising that we only discovered one (1%) mutation among our NSCLC cases. In malignant melanoma, the frequency of *BRAF* mutations is higher (66%) and the majority (80%) are accounted for by a single substitution in codon 599 converting valine (V) to glutamic acid (E) 39. Single substitution in V599 increases the kinase activity of B-Raf, rendering the MAPK pathway constitutively active. Other mutations in *BRAF* also confer increased kinase activity. However, the ratio between V599 and non-V599 mutations seems to be the opposite in lung cancer compared to malignant melanoma 37–40. Here, we revealed one D593A mutation, that is non-V599. The amino acid at position 593 belongs to the conserved DFG motif and substitution of aspartic acid to alanine appears to inhibit the kinase activity of B-raf 39,41. It has been suggested that the *BRAF* D593V, which is believed to have the same function as D593A, does not primarily contribute to carcinogenesis. It is rather the *KRAS* mutations, which D593V often coincides with, that contribute to carcinogenesis 41. In our study, D593A does not coincide with a *KRAS* mutation but instead with a mutation of the PIK3CA. As D593A does not appear to confer with an increased activity of B-Raf, it is likely that it is a so-called passenger mutation 42. Passenger mutations are incidental; they do no infer growth advantage and are not the subject of selective pressure, as opposed to the driver mutations. It is estimated that the majority of all mutations are passengers and it is important to distinguish them from the true drivers, which confer an advantage for the cell in which they occur.

In this study, we were not able to find any association between *IRS-1* G972R and risk of developing NSCLC. This might reflect the limited number of cases and the small groups they produce upon stratification. The *IRS-1* G972R polymorphism has earlier been correlated to risk of developing prostate 43, colon 44, and breast cancer 45, however, there are no published studies on NSCLC. In all three cases, the R allele increased the risk of developing cancer. On the other hand, in the study on breast cancer the increased risk was only seen among Hispanic women, whereas a study conducted by Wagner et al. 46, in which Polish familiar breast cancer cases were enrolled, no association was observed between the *IRS-1* G972R polymorphism and risk of breast cancer. Even though no correlation was found in our case, there might still be other mechanisms regulating, for example, the expression of *IRS-1*, possibly through other SNPs within the gene or promoter, which might affect the lung carcinogenesis. However, the impact of low-penetrance variants (like SNPs) can be disguised by high-penetrance mutations 47. This has been seen in colorectal cancer where the *MDM2*-SNP309 was hypothesized to act as a low-penetrance gene and stratification was performed according to *P53* mutation status. No association was found between SNP309 genotype and the median age at diagnosis when all individuals regardless of *P53* status were included. However, excluding individuals with mutated *P53* or unknown *P53* status revealed a statistically significant association between the age at colorectal cancer diagnosis and *MDM2*-SNP309. These results show that low-penetrance variants can have important oncogenic roles and that it is important to distinguish them among the high-penetrance mutations. However, according to our results, *IRS-1* G972R does not seem to act as a high-nor low-penetrance variant in NSCLC and does not confer with an increased risk of developing NSCLC.

In conclusion, we have shown that crucial components of the PI3K and MAPK pathway discriminate between NSCLC subtypes. Especially interesting is that several components of the PI3K pathway, alone and in combination, are correlated to development of SCC of the lung.
